# Draft Genome Sequence of the Yeast *Starmerella bombicola* NBRC10243, a Producer of Sophorolipids, Glycolipid Biosurfactants

**DOI:** 10.1128/genomeA.00176-15

**Published:** 2015-03-26

**Authors:** Tomohiko Matsuzawa, Hideaki Koike, Azusa Saika, Tokuma Fukuoka, Shun Sato, Hiroshi Habe, Dai Kitamoto, Tomotake Morita

**Affiliations:** a Research Institute for Innovation in Sustainable Chemistry, National Institute of Advanced Industrial Science and Technology (AIST), Tsukuba, Ibaraki, Japan; b Bioproduction Research Institute, National Institute of Advanced Industrial Science and Technology (AIST), Tsukuba, Ibaraki, Japan

## Abstract

The yeast *Starmerella bombicola* NBRC10243 is an excellent producer of sophorolipids (SLs) from various feedstocks. Here, we report the draft genome sequence of S. bombicola NBRC10243. Analysis of the sequence may provide insight into the properties of this yeast that make it superior for use in the production of functional glycolipids and biomolecules, leading to the further development of S. bombicola NBRC10243 for industrial applications.

## GENOME ANNOUNCEMENT

The ascomycetous yeast *Starmerella bombicola* produces large amounts of sophorolipids (SLs) that consist of a sophorose head group (2-O-β-d-glucopyranosyl-d-glucopyranose), the anomeric carbon atom of which is attached to an ω- or (ω1)-hydroxylated C18 or C16 fatty acid. Massive amounts of SLs are produced by S. bombicola from feedstocks as a mixture of lactone-form and acid-form SLs (1, 2). SLs and their derivatives show surface-active and emulsification properties, as well as other beneficial properties such as antimicrobial, anticancer, and antiviral activities, and are useful in the food and detergent industries (3).

Here, we describe the draft genome sequence of S. bombicola NBRC10243 (=ATCC 22214) as a typical SL producer. A paired-end DNA library of S. bombicola NBRC10243 genomic DNA was sequenced using the MiSeq system (Illumina) to approximately 300-fold coverage, comprising a total of 11,269,740 reads with a length of 2 × 250 nucleotides and an insert size of approximately 300 nucleotides. Running the SOAPdenovo assembler with a k-mer size of 63 (4) using the genomic fragments generated an assembly of 296 contigs (≥1,000 bp), resulting in 9.37 Mb for the whole S. bombicola genome (longest contig, 432 kb; shortest contig, 1.0 kb; *N*_50_, 87.6 kb) with a G+C content of 48.1%.

Protein coding genes were automatically predicted by Augustus (5) using Saccharomyces cerevisiae, and the models created resulted in a 4,599 protein-coding gene set. The gene cluster responsible for SL biosynthesis in S. bombicola (6) was identified on the two scaffolds. Two oligonucleotide primers, 5′-CTCACACACAACGATTGCAGTATATTTACC-3′ and 5′-CAGGATCGGGCCCTCGCTCCAATGAATAAC-3′, were prepared to amplify the gap region between the two contigs. The obtained gene fragment was sequenced using the conventional Sanger method, after which the two contigs were connected to conserve the complete length of the gene cluster for SL biosynthesis. The first step for SL biosynthesis is terminal hydroxylation of a fatty acid by the reaction of a cytochrome P450 monooxygenase (CYP52M1). SL is then formed via the reactions of two glucosyltransferases (UGTA1 and UgTB1) and an acetyltransferase (AT) and is secreted by an ABC transporter (MDR) (7). The five CDSs of the cluster were identical to *ugtb1* (HM440974), *mdr* (HQ660581), *at* (HQ670751), *ugta1* (HM440973), and *cyp52m1* (EU552419), respectively. This genome sequence will provide a novel aspect for the use of S. bombicola as a platform organism for the production of various biomolecules.

### Nucleotide sequence accession numbers.

The nucleotide sequence of the S. bombicola genome has been deposited in DDBJ/EMBL/GenBank under the accession numbers BBSW01000001 to BBSW01000295 (295 entries).
